# The York Gospels: a 1000-year biological palimpsest

**DOI:** 10.1098/rsos.170988

**Published:** 2017-10-25

**Authors:** Matthew D. Teasdale, Sarah Fiddyment, Jiří Vnouček, Valeria Mattiangeli, Camilla Speller, Annelise Binois, Martin Carver, Catherine Dand, Timothy P. Newfield, Christopher C. Webb, Daniel G. Bradley, Matthew J. Collins

**Affiliations:** 1 Smurfit Institute of Genetics, Trinity College Dublin, Dublin 2, Ireland; 2 BioArCh, University of York, York YO10 5DD, UK; 3 Department of Archaeology, University of York, York YO10 5DD, UK; 4 Borthwick Institute for Archives, University of York, York YO10 5DD, UK; 5 Department of Preservation, The Royal Library, København K DK-1016, Denmark; 6 Department of Archaeology, University of Paris 1 Panthéon-Sorbonne, 3 rue Michelet, 75006 Paris, France; 7 Departments of History and Biology, Georgetown University, 37th and O Streets NW, ICC 600, Washington, DC 20057, USA; 8 Museum of Natural History, University of Copenhagen, Copenhagen, Denmark

**Keywords:** molecular codicology, ancient DNA, parchment, microbiome, eZooMS

## Abstract

Medieval manuscripts, carefully curated and conserved, represent not only an irreplaceable documentary record but also a remarkable reservoir of biological information. Palaeographic and codicological investigation can often locate and date these documents with remarkable precision. The York Gospels (York Minster Ms. Add. 1) is one such codex, one of only a small collection of pre-conquest Gospel books to have survived the Reformation. By extending the non-invasive triboelectric (eraser-based) sampling technique eZooMS, to include the analysis of DNA, we report a cost-effective and simple-to-use biomolecular sampling technique for parchment. We apply this combined methodology to document for the first time a rich palimpsest of biological information contained within the York Gospels, which has accumulated over the 1000-year lifespan of this cherished object that remains an active participant in the life of York Minster. These biological data provide insights into the decisions made in the selection of materials, the construction of the codex and the use history of the object.

## 1. Introduction

Medieval manuscripts are objects of great worth and value, often in the past elaborately decorated and bound, emphasizing their importance, not only as literary texts but also as physical objects of intrinsic and spiritual value, as well as tangible examples of traditional craft production. Moreover, a contemporaneous collection of animal skins bound together provides a remarkable biological resource, which may allow for insights into the husbandry of the source animals and in turn shed light on the assembly of the codex. The utility of parchment documents as a store of biological information is confirmed by a number of molecular studies, which have successfully retrieved DNA sequences from parchments and produced comparisons with modern reference populations of cattle, sheep and goats [1–4]. These analyses have used isolated parchment fragments, which are then digested as part of the DNA extraction process. Such studies have yet to include bifolia from bound volumes, possibly due to this requirement for destructive sampling.

In the light of the vast potential afforded by medieval manuscripts, several authors are experimenting with non-destructive sampling of documents. While most of these methods involve some form of spectroscopic analysis, novel approaches, which release molecules from the surface, such as the use of synthetic gel films [5,6] have been reported, and this work can be seen as part of a wider push to develop sampling methods for material culture [7–11]. These studies also form part of an increasingly sophisticated analysis of an object's preservation status, which in the case of parchment, includes concerns over both deterioration and risks to personnel health caused by microbial contamination [6,12–14].

Analyses of the traces left by the handling and use of an object are common in Palaeolithic and Neolithic archaeology and have been applied to personal adornment artefacts (e.g. [15,16]) as well as stone tools. Much less common in the historical period, they can illuminate the choices made in the selection of raw materials, and the life history of an object [17]. A pioneering application of this analysis is Kate Rudy's [18] use of densitometry to map the discoloration of parchment caused by repeated usage. Her work highlights a third level of biological data, the grime on the surfaces of parchments that attests to handling. In some cases, folia have experienced much more active intervention, such as devotional kissing or rubbing, activities that are likely to leave distinct microbial traces, which can be explored using biomolecular methods.

The analysis of both the raw materials of the codex (i.e. the skins selected from flocks and herds), and the microorganisms on the object (which may highlight its use history and conservation risk) must be compatible with conventional conservation treatment. Our contribution to this field has been to develop [19] and here to refine a triboelectric (eraser-based) sampling method, to recover first protein and now DNA from parchment. Dry cleaning with PVC erasers is a common and widely used conservation technique that allows the surface of the parchment to be cleaned without causing damage to the object [20], and we analyse the waste material from this process, which would otherwise be discarded.

We apply our approach to document for the first time the vast array of biological information contained within a single codex, recovering both DNA and proteins from the dry eraser waste of skins from the York Gospels (electronic supplementary material, York Minster Ms. Add. 1). One of only a small collection of pre-conquest (1066 CE) Gospel books to have survived the Reformation, the York Gospels are thought to have been written around the turn of the first millennium in the scriptorium of St Augustine's monastery in Canterbury [21] and then brought to York by Archbishop Wulfstan *ca* 1020 CE. The text contains all four gospels of the New Testament; a letter from King Cnut (r. 1016–1035 CE) and documents concerning land ownership. It is the only surviving English Gospel book to contain the oaths taken by the deans, archdeacons, canons and vicars choral, dated to the fourteenth to sixteenth centuries, and is still used in ecclesiastical ceremonies today. Our study presents the rich palimpsest of biological information housed within this document, which we then compare to six legal documents with different curatorial histories from two collections held at the Borthwick Institute for Archives (Borthwick Archive).

## 2. Results

### 2.1. Species identification

#### 2.1.1. Protein analysis

**Figure 1.**
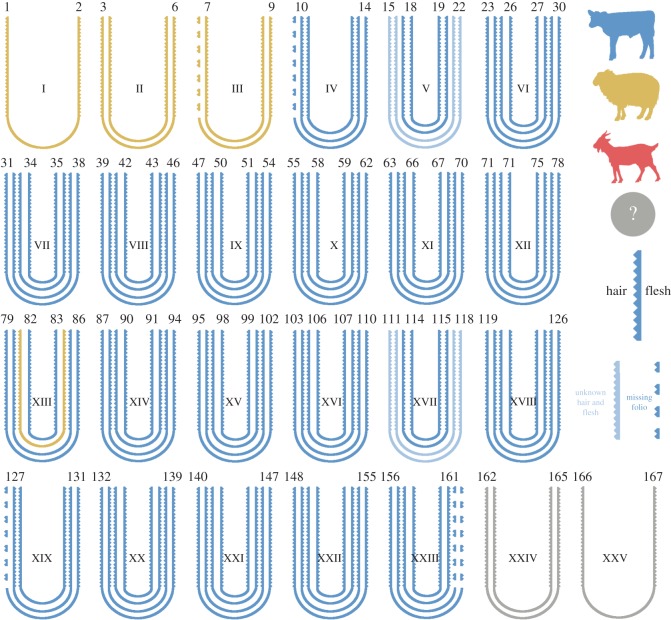
Structure of the quires comprising the manuscript, and species identification of the bifolia using eZooMS.

This is the first systematic application of eZooMS [19], a non-invasive method of species identification through mass spectrometry, to identify the source species of every bifolium contained within a single codex (electronic supplementary material, table S1). The York Gospels were found to be composed of two animal species: the original gospels are of calfskin (except for one bifolium made of sheepskin) and the later additions (fourteenth century) are made exclusively of sheepskin (figure 1; electronic supplementary material, figure S1). Owing to extensive conservation treatments carried out in the 1950s that involved covering entire folia in silk gauze, we were unable to determine the species for the final quire and flyleaves (folia 162–167).

#### 2.1.2. DNA analysis

DNA extraction was attempted from eight bifolia of the York Gospel from which large volumes of eraser waste (150–250 µl) had been generated; sufficient DNA concentrations were recovered from all samples for successful library preparation and high-throughput DNA sequencing. Initial species identification was undertaken with FastQ Screen (a tool to informatively screen DNA sequences against multiple genomes) and in all cases, the genetic species assignments agreed with those produced through eZooMS (electronic supplementary material, table S2). One sample (folio 158, Quire XXIII), which had previously provided an inconclusive species identification of calf during the proteomic analysis was also assigned as calf in the genetic analysis. However, this page is one of those that suffered extensive invasive conservation, and even this combined assessment remains somewhat tentative. Insufficient data were recovered from two samples taken from the fourteenth century additions to the Gospels to confidently assign the source species using solely genetic methods; however, both samples were previously conclusively identified as sheep via eZooMS.

### 2.2. Genetic analysis

DNA sequences recovered from the parchment samples were stringently (electronic supplementary material, table S3) aligned to the genome of the identified host (production) species to estimate the proportion of endogenous (source species) DNA retained within the bifolia of the York Gospels (electronic supplementary material, methods). This analysis resulted in a mean endogenous percentage of 19.3% (range 0.7–51.4%) over all the samples (electronic supplementary material, table S3). For folio 125 (quire XVIII), 51.4% of reads could be aligned to the genome of the source species (cow); however, this assignment fell to 5.6% when filters for read mapping quality were applied (electronic supplementary material, table S3). While some loss of mapped reads is expected post filtering, this extreme reduction suggests that there may be a bias in DNA sequence preservation or retrieval from the manuscript, one that in this case favoured repetitive genomic regions over more gene-rich euchromatic regions. This apparent taphonomic bias may be a reflection of the harsh alkaline treatment that is an integral part of the parchment production process, which might selectively degrade the more loosely packed euchromatin over the tightly bound heterochromatin (electronic supplementary material, figure S2).

A mapDamage2.0 [22] analysis was completed on the filtered host reads recovered from the York Gospels to explore DNA damage patterns and recovered DNA authenticity (electronic supplementary material, figure S3). All samples were found to have characteristic markers of degraded ancient DNA (aDNA) with an increase in deamination at the end of reads. However, the frequency of these modifications is much lower than what would be predicted for bone of a similar age [23]. Given that the DNA fragment lengths are also short in these recovered sequences (electronic supplementary material, figure S4), this result may suggest that different DNA degradation processes are active in parchment and bone.

Sufficient sequence data were recovered from three samples (Fol. 13, Fol. 101 and Fol. 125) to permit further population genetic analyses using genome-wide single nucleotide polymorphisms (SNPs). These samples were placed onto a reference dataset of modern cattle [24] in a principal component analysis (PCoA) (electronic supplementary material, figure S5*a*,*b*) using the LASER2.0 software [25,26]. Although only a relatively small number of variants could be called in each of the three samples, they can be seen to cluster with modern cattle breeds of Northern Europe (electronic supplementary material, figure S5*b*). Specifically, the sample with the highest genomic coverage, folio 101 (2139 bovine HD SNPs called) falls just outside a cluster of Norwegian red and Holstein animals, with the two lower coverage samples, folio 13 and folio 125, falling outside of the major European distribution (electronic supplementary material, figure S5*b*). The position of folio 13 and 125 probably reflects the limited SNP recovery in these samples; however, it could also be an indication of true genetic diversity in these animals. As further studies of geographically and temporally localized animal bone reveal the genetic landscape of medieval cattle, we will be in a stronger position to localize these and future parchment objects.

Sex identification [27] was attempted for all eight bifolia sampled for DNA analysis (electronic supplementary material, table S2), with high confidence assignments being deduced for five. Four out of the five reliably typed animals in the original Gospel document (prior to the later *ca* fourteenth century additions) were found to be female.

### 2.3. Exogenous DNA

#### 2.3.1. Human

As the novel non-invasive DNA sampling technique used in this study samples molecules from the surface of documents, the exogenous/environmental DNA residing on the York Gospels was explored. Firstly, to discern an estimate for the upper bounds of human DNA on the document, next-generation sequencing reads recovered from the Gospels were aligned to the human genome (hg19). The average recovery of human DNA sequences over the whole document was 11.1% (range 4.2–20.8%) (electronic supplementary material, table S4), almost twice that of six control samples—legal documents (title deeds) held in the Borthwick Institute for Archives—sampled using identical methods (average 5.6%, range 1.9–12.8%). This lower proportion of human DNA residing on the control samples may reflect the use history of the documents, and the less intensive handling of legal deeds compared to the York Gospels. Sequences that aligned solely to the human genome show a reduction in DNA damage patterns compared to those from the host animal skin itself (electronic supplementary material, figure S3), potentially indicating their more recent origin from ongoing handling. However, the overall DNA fragment lengths of the endogenous and exogenous molecules are comparable, with a possible slight shift to lower fragment lengths in the endogenous (parchment) molecules (electronic supplementary material, figure S4).

For two York Gospel samples (Fol. 6 and Fol. 158) over 19% of recovered reads were mapped to the human genome (electronic supplementary material, table S5); this dropped to 16% when filters for mapping quality were applied (electronic supplementary material, table S5). The reduction in alignment percentage due to mapping quality filtering is not as dramatic as that seen in the endogenous (parchment) DNA, a result consistent with the idea that these molecules were deposited after parchment manufacture. Folio 158 (15.3%) was the subject of extensive and invasive conservation treatment in the last century and the exogenous DNA fragment lengths from this parchment show striking periodicity consistent with histone wrapping, [28,29] a result that may be indicative of a significant amount of DNA degradation (electronic supplementary material, figure S4). Folio 6 (17.5%) contained the ‘Oaths of a subdean in person, a canon and prebendary in person, and an archdeacon’, passages which we would anticipate to be more regularly read/handled than the other folia studied, consistent with the greater level of human DNA recovered from this page.

#### 2.3.2. Microbiome

To further classify the York Gospels metagenome, recovered sequences were analysed using two independent metagenomic pipelines, One Codex [30] and metaBIT [31] (electronic supplementary material, figure S6*a*,*b*) to try to reduce method-based classification biases [32]. Six samples from archival documents held in the Borthwick Institute for Archives were also included for comparison. The taxon distributions generated from the metaBIT and One Codex analysis of the York Gospel samples were found to be consistent with those previously reported for the skin microbiome (electronic supplementary material, figure S6*a*,*b*) [31,33]. This result is in agreement with the fact that the skin microbiome is the most common component of the urban microbiome [34], in particular on handled surfaces [35].

**Figure 2.**
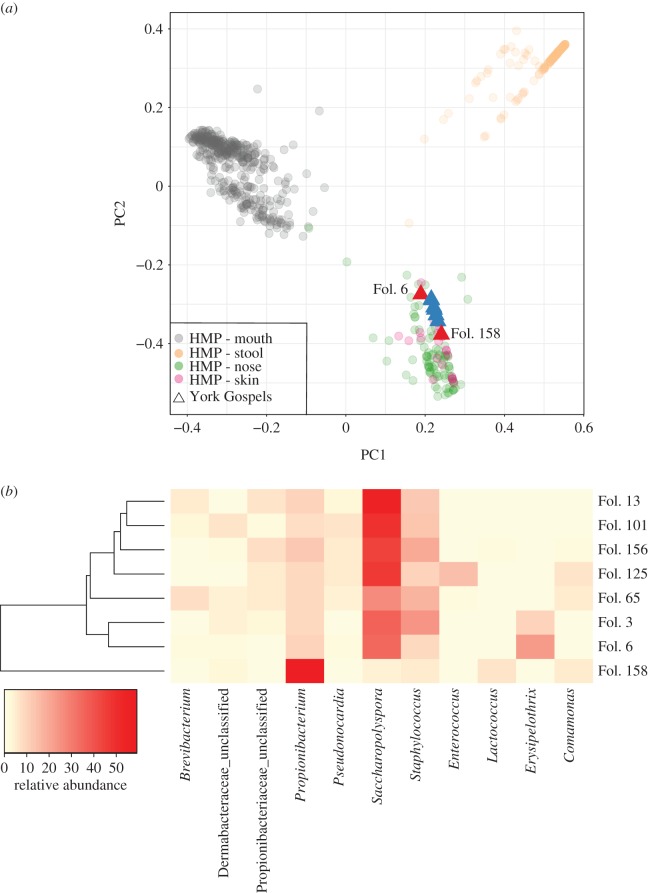
Metagenomic analysis of the York Gospel samples (*a*) PCoA of York Gospels samples (host filtered) at the genus level analysed with a human microbiome project (HMP) background. The two York Gospel samples with the greatest concentration of human DNA (Fol. 6 and 158) are highlighted in red. (*b*) Heat map of microbial genus relative abundance in each of the York Gospel samples; genera shown represent greater than 5% abundance in at least one sample. Clustering of samples (dendrogram) was completed using the complete metaBIT genus filtered output. The highly conserved sample Fol. 158 is seen as an outlier to the other York Gospel samples and the two later additions (Fol. 3 and 6) are seen to cluster.

To further investigate this skin microbial signature, a PCoA was performed. This analysis combined the York Gospel samples with the human microbiome project (HMP) human-associated microbial profiles provided with metaBIT (genus level, figure 2*a*). All the York Gospel samples irrespective of their human/endogenous DNA percentages or conservation status were found to have a microbial profile which placed them within the HMP skin and nose diversity (figure 2*a*). This tight clustering of the York Gospel samples is not seen in the six comparative documents, whose broader distribution may reflect their more diverse life histories prior to and within the archives (electronic supplementary material, figure S7). The skin microbial signature is further highlighted in an abundance heat map (figure 2*b*), which shows the presence of skin microbial markers (e.g. *Propionibacterium* and *Staphylococcus*) at high relative frequencies. Importantly, these skin microbiome signatures are absent in the control sample (electronic supplementary material, figure S8), suggesting they reflect microbiota colonizing the parchment itself and are not the result of laboratory contamination [36,37]. Of importance to the continued conservation of the York Gospels is the discovery of the *Saccharopolyspora* genus on all bifolia including the conserved sample folio 158. This bacterial genus has been identified previously by Piñar *et al.* [13] as a possible cause for a measles-like (maculae) spotting of parchment, which is associated with localized collagen damage and document degradation [13].

To explore shared patterns of microbial colonization within the York Gospels, samples were clustered according to their genus profiles (dendrogram, figure 2*b*). This analysis placed the microbial composition of the highly conserved folio 158 as an outlier to all other York Gospel samples; moreover, the two later additions to the manuscript (Fol. 3 and Fol. 6) are seen to cluster. Interestingly, two samples with relatively high endogenous DNA content (Fol. 13 and 101) also fall together, a tentative hint to the possibility of future correlations between microbial colonization and DNA retrieval. The clustering analysis was then repeated using the combined Borthwick Archive/York Gospels dataset (electronic supplementary material, figure S8). In this analysis three major groupings can be seen; firstly, folio 158 is again seen as an outlier. Two further internal groupings are then revealed (electronic supplementary material, figure S8): one composed of the non-conserved York Gospel samples and two Borthwick Archive samples (BA39 and BA74), and a second containing the remaining Borthwick Archive samples (4/6).

To investigate the relationship between the metagenomes of the York Gospels and the Borthwick Archive samples further, STAMP [38] was used to profile a MetaPhlAn2 analysis (electronic supplementary material). In a PCoA analysis of the samples at the genus level, BA39 and BA74 were again seen to cluster with the non-conserved York Gospel samples, away from the four other Borthwick Archive samples (electronic supplementary material, figure S9*a*). When BA39 and BA74 were directly compared to the remaining Borthwick Archive samples again with STAMP (Welch's *t*-test), three taxa (electronic supplementary material, figure S9*b*) were significantly differentiated between the two groups (*Granulicella*, *Saccharopolyspora* and *Pseudonocardia*). The increased prevalence of *Saccharopolyspora* in BA39 and BA74 may therefore indicate why these documents cluster with the York Gospel samples given the significant presence of this genus on the manuscript (electronic supplementary material, figure S9*b*).

A final comparison was conducted in STAMP (Welch's *t*-test) between the filtered Borthwick Archive dataset (BA39 and BA74 removed) and the non-conserved York Gospel samples (electronic supplementary material, figure S9*c*), highlighting five significantly differentiated taxa between the groups (*Saccharopolyspora*, *Pseudonocardia*, *Actinopolyspora*, *Propionibacterium* and *Staphylococcus*). These results seem to further reflect the differences in the level of handling between the documents, with the York Gospels microbiome containing significantly more of the skin microbiome components *Propionibacterium* and *Staphylococcus*, but might also provide insights into their relative conservation priorities with the York Gospels being more heavily colonized overall by *Saccharopolyspora* (electronic supplementary material, figure S9*c*).

The limited number of significantly differentiable taxa in these metagenome comparisons may be indicative of the small sample size in this study and the inherent difficulty of metagenomic taxonomic assignment from shotgun sequencing [32]. However, it could also be an indication of a common parchment microbiome, maintained by the specific microbial growth conditions (salt-rich) provided by the parchments' surface [13].

## 3. Discussion

Medieval manuscripts represent an irreplaceable historical record, but the need to conserve these documents is seemingly at odds with their value as an important reservoir of contemporaneous biological information. By extending the non-invasive eZooMS method to include the analysis of DNA, we propose a cost-effective and simple-to-use biomolecular sampling technique to enable this historical resource to be explored. The current study applies this combined methodology to document for the first time a rich palimpsest of biological information from a complete book object of great cultural value over its 1000-year history.

### 3.1. Species composition and book production

Within this study, the species composition of the York Gospels has been revealed; the primary document is composed almost exclusively of calfskin, apart from a single bifolium made of sheepskin. This detailed analysis further highlights the utility of eZooMS for high-throughput species identification of manuscripts. With low cost and analysis time per sample, this technique has the potential to describe the species composition of many other documents and provide further insights into medieval manuscript production.

Zooarchaeology usually struggles to obtain accurate population-sized assemblages, with collections often processed and fragmented and rarely constrained to a narrow time range [39,40]. The analysis of the species composition of manuscripts may therefore have implications, which extend beyond the documents' production, by providing a more refined understanding of past animal population sizes with a tighter chronology than can often be obtained from archaeological assemblages alone.

Although a possible artefact of the small sample number, the frequency of female animals (4 females, 1 male) among the calves is worthy of mention. As cattle are slaughtered for parchment production as juveniles (electronic supplementary material), male calves, of lesser reproductive value than females, would be hypothesized to be most often selected for this purpose. If this contradictory pattern of an excess of female calves were to be confirmed by further sampling, a possible explanation could lie in the correlation between the writing of the manuscript and a historical outbreak of murrain, tentatively identified as rinderpest or a closely related morbillivirus ancestor. Although the composition date of the York Gospels is still debated, it is possible they were written in Canterbury around 990 CE, shortly after a major outbreak of cattle plague occurred in the British Isles. At least seven independent sources describe widespread cattle mortalities in England, Wales, Ireland and possibly Scotland between 986 and 988, with medieval and later texts documenting that the flaying of the carcass of a diseased animal to employ its skin was an accepted way of cutting losses [41,42]. The murrain could therefore have produced an abundant source of fetal and newborn calfskins of both sexes that were possibly so numerous that they were still being used as parchment 2–4 years after the outbreak. Indeed, even if these skins were not produced as a consequence of the murrain but there was a widespread local mortality in the years before the text was written, it would seem unusual to sacrifice the very animals (females) required to rebuild the herd. Further analyses of the mortality patterns and local outbreaks of disease could help to refine the chronology of the text.

An alternative explanation could lie in the value of the female calves themselves, as cattle are proposed to be a great source of wealth and power in the Anglo-Saxon world. If female calves are of higher value than male, perhaps the selection of female animals reflects the choice of the very best and most expensive material available to receive the holy word: a sacrifice of prized animals fit to answer the sacrifice of the Lord, and to demonstrate the faith and the wealth of the commissioner of the manuscript [43].

Finally, our assumption about the higher value of female calves, which relies mostly on data from earlier (Roman period) or later (thirteenth to fourteenth century) texts, may be erroneous. The Saxon economy relied mostly on oxen for traction [44], and the latter may have out-valued heifers, leading to occasional surfeits of female calves, which could be used for parchment production.

In this context of the perceived value of calves, the presence of a single sheepskin bifolium in the original Gospel text seems out of place. In the accounts of a Cistercian Abbey at Beaulieu (1269–1270), the best sheepskin parchment is worth less than the worst calf parchment [45]. Determining the extent to which the selection of sex and of species reflected differences in quality, perceived notions of value or a spiritual dimension would require a more comprehensive study of Anglo-Saxon manuscripts.

In relation to the later sheepskin additions to the document, a sixteenth-century inventory describes the York Gospels as ‘A text, decorated with silver, not well gilt, on which the oaths of the dean and other dignities and canons are inserted at the beginning’ [21]. This description would seem to imply that not only the text but the bifolia themselves were later fourteenth century additions. A possible explanation for the use of sheepskin for these subsequent additions that contain oaths, deeds and personal correspondence is found in the *The Dialogus de Scaccario* [46], which describes a preference for legal documents to be written on sheepskin to avoid erasure and fraud of the written details. Unlike calf and goat, sheepskin has a lower density of collagen fibres at the base of the (more abundant) hair follicles, which means that the skin can split and the upper layer peel away if the parchment is roughly abraded.

### 3.2. Population genetic analysis

This study is the first to our knowledge to use a non-invasive method to retrieve host genetic data from parchment, arguably the most important biological material of the Medieval world. We were able to recover endogenous DNA from all six of the samples taken from the original, almost 1000-year-old York Gospel document. Although only limited DNA sequencing was undertaken in this analysis, there were still sufficient data to estimate the genetic affinities of half of these samples, placing them within European cattle diversity.

The loss of more data than would be expected through standard bioinformatic filters for repetitive sequences may represent a limitation to this nucleotide retrieval technique, as the abundance of these sequences found in our dataset restricted the utility of the host genomic data. However, this increase in repetitive sequences has also been observed to a lesser extent in DNA samples from younger cut pieces of sheep parchment [1]. Given the extensive sampling opportunities that could be provided by our novel non-invasive method, a further comprehensive analysis of a diverse range of documents is warranted to see if this effect is truly a consistent artefact of eraser-based extraction, or an as yet undescribed feature of DNA recovered from older parchments. Encouragingly, one sample (folio 101) did have sufficient genetic density to enable clear comparisons with reference populations and was found to closely resemble breeds of northwest Europe, a result that matches the provenance of the object.

These analyses highlight a second layer of information contained within medieval manuscripts, which speaks to the historical management of animals and the further possibility of geolocating the source of the parchment, by comparison with the archaeological record of butchered bone. Moreover, as methods for DNA recovery and analysis are optimized further, the phenotype of animals selected for parchment production may be documented [47,48]. Genetic analyses could reveal not only the sex of the animals, but also their coat colour and morphological features (polled versus non-polled), while epigenetic analyses may in the future provide insight into the animal's age [49,50].

### 3.3. Metagenomics

The analysis of microbial communities that inhabit the built environment is an expanding field of research, and is moving into heritage science. We have developed an easy-to-use non-invasive sampling method, which can recover high-resolution microbial data from sensitive documents. Our culture-free technique has benefits over other recently proposed sampling methodologies [6] in that it co-opts a widely accepted manuscript-cleaning technique [20] and as such can be reliably implemented into workflows by conservators and codicologists.

One of the greatest factors influencing interpretation of microbiomes is technical variation in the analysis [51]; we, therefore, opted for a shotgun approach to guard against known artefacts in the 16S analysis of short aDNA sequences [52]. Moreover, although it is known that HMWt DNA is preserved on filter paper [53] (a surface in some ways analogous to parchment), we chose not to shear the extracted DNA, in an attempt to exclude recent DNA transferred by handling and preserve highly degraded endogenous molecules [54]. Reagent contamination is also a known issue for studies such as ours with limited DNA starting concentrations [36,37], which we addressed through the use of appropriate blank controls. Finally, we used multiple software packages in our analysis, to improve the resolution of taxon presence and abundance [32]. Despite the relative immaturity of shotgun metagenomic analyses, it is encouraging that the different analytical pipelines used in the study gave relatively consistent results, and the species of interest have overlaps with those previously described in the parchment microbiome.

The microbial signature of the York Gospel reflects the nature and use of this document in that it resembles that of the human skin microbiome, reiterated by the level of human DNA discovered on the surface of the document with values of over 15% of recovered reads in some cases. The increased abundance of the *Propionibacterium* genus seen in this analysis compared to other studies, probably reflects the significant amount of handling the York Gospels has been subjected to, including its use in ecclesiastical ceremonies to this day. An alternative explanation is that the true extent of colonization by *Propionibacterium* species on parchments may have been underestimated by some 16S studies as the targeted sequencing of the hypervariable region 4 has been shown to limit the resolution of skin commensal microbiota, particularly *Propionibacterium* [55]. The discovery of the possibly destructive *Saccharopolyspora* genus [13] at high concentrations on the surface of the York Gospels further highlights the utility of metagenomic analyses that seek to describe the parchment metagenome. Medieval manuscripts are part of our collective heritage and methods like these that can target conservation efforts will be of great use.

Comparison of the microbial signatures generated in this analysis highlighted a distinct difference between a highly conserved bifolium and the remaining bifolia from the York Gospels and enabled documents that were later additions to the manuscript to be distinguished. These results raise the possibility of future molecular provenancing of documents as more manuscripts have their microbiomes explored. The metagenomic signals from our comparisons of the Borthwick Archive and York Gospel samples strengthen this assertion as, overall, we could distinguish between the two groups. The limited number of statistically differentiated taxa between these sample sets with very divergent conservation histories raises the possibility that the microbial colonization of documents though widespread may be limited to certain species that can tolerate the growth conditions at the parchments' surface [13].

## 4. Conclusion

This study is the first non-invasive biomolecular analysis of a complete book object. Both protein and genetic analyses have been applied to reveal the animal origins of the 167 parchment folia that make up the York Gospels. This is the first time a non-invasive sampling technique has been used to recover host DNA from parchment manuscripts, which has allowed not only for the determination of source species but also an estimate of the genetic affinities and sex of the animals with limited sequencing. In addition, our novel sampling method enabled the recovery of detailed information on the microbiome associated to individual folia, which not only will be of great interest for book conservation, but also has the potential to reveal past storage conditions and use of book objects.

## 5. Material and methods

The York Gospels and archival documents were sampled using the dry non-invasive eraser-based sampling technique of Fiddyment *et al.* [19], with 86 folia sampled for protein analysis, and a further eight folia and six archival documents sampled for DNA analysis. eZooMS analysis of the York Gospels and archival documents was completed following the protocol of Fiddyment *et al.* [19]. DNA was extracted following the protocol of Fiddyment *et al.* [19] with a single modification: the use of eraser crumbs (*ca* 150–250 µl) in place of cut parchment samples (electronic supplementary material). Illumina sequencing libraries were produced for each of the samples and appropriate controls following the protocol of Meyer & Kircher [56] as modified by Gamba *et al.* [57] and sequenced on an Illumina MiSeq. Raw sequencing reads were trimmed of adapter sequences using cutadapt [58], screened with FastQ Screen and aligned to appropriate reference genomes using BWA [59] and then filtered with SAMtools [60]. DNA damage assessments were completed using mapDamage2.0 [22] and population genetic PCoA analyses with LASER2.0 [25]. Metagenomic analyses were completed from host filtered datasets (electronic supplementary material) using metaBIT [31], One Codex [30] and STAMP [38].

## Supplementary Material

Electronic Supplementary Material - The York Gospels: a one thousand year biological palimpsest

## Data accessibility

Raw Illumina sequence data are deposited at the European Nucleotide Archive http://www.ebi.ac.uk/ena/data/view/PRJEB21551; MALDI-TOF data are deposited at the Dryad Digital Repository (http://dx.doi.org/10.5061/dryad.1p390) [61].
