# Supplementary material for: The York Gospels: a 1000-year biological palimpsest
Source: R Soc Open Sci. 2017 Oct 25;4(10):170988. doi: 10.1098/rsos.170988 (PMC5666278; doi:10.1098/rsos.170988)
Supplement: Electronic Supplementary Material - The York Gospels: a one thousand year biological palimpsest [file rsos170988supp1.pdf]

The York Gospels: a 1000-year biological palimpsest

Authors

Matthew D. Teasdale[1*+], Sarah Fiddyment[2*+], Jiří Vnouček[2,3], Valeria Mattiangeli[1], Camilla Speller[2], Annelise Binois[4], Martin Carver[5], Catherine Dand[6], Timothy P. Newfield[7], Christopher C. Webb[6], Daniel G. Bradley[1] and Matthew J. Collins[2,8+]

1. Smurfit Institute of Genetics, Trinity College Dublin, Dublin 2, Ireland.
2. BioArCh, University of York, York, YO10 5DD, UK.
3. Department of Preservation, The Royal Library, DK-1016 København K, Denmark
4. Department of Archaeology, University Paris 1 Panthéon-Sorbonne, 3 rue Michelet, 75006 Paris, France.
5. Department of Archaeology, University of York, York, YO10 5DD, UK.
6. Borthwick Institute for Archives, University of York, York, YO10 5DD, UK.
7. Departments of History and Biology, Georgetown University, 37th and O Streets NW, ICC 600, Washington, DC, USA, 20057.
8. Museum of Natural History, University of Copenhagen, Copenhagen, Denmark.

* These authors contributed equally to this work.
+Corresponding authors m.teasdale@tcd.ie, sarah.fiddyment@york.ac.uk and matthew.collins@york.ac.uk

Table of contents

1. Parchment samples **2**
The York Gospels 2
Estimation of the size of the animal skin 2
Borthwick Archive documents 2
2. Biomolecular analysis **3**
Parchment sampling 3
DNA extraction, library preparation and sequencing 3
Read processing and analysis 3
Read filtering 4
DNA damage assessment 4
Sex determination 4
Population genetic analysis 4
Metagenomic analysis 4
STAMP analysis 5
3. Supplementary Figures **6**
4. Supplementary Tables **15**
5. References **22**

Supplementary Materials and Methods

1. Parchment samples

The York Gospels

Document statistics

York Gospels: York Minster Ms. Add. 1 (York Minster Library).

Dimension of folio: 270 x 207 mm.

Dimension of bifolium: 412 x 270 mm (mostly one bifolium per skin).

Number of folia: 167 (original Gospel 152).

Quires: 25 (original Gospel 20 all of them are quaternions – 4 bifolia in one quire).

Hair/flesh orientation of all quires: H|F, F|H, H|F, F|H|F, F|H, H|F, F|H.

Animals: sheep and calf (in original Gospel only one sheep bifolium 81/84).

Estimation of the size of the animal skin

The estimated usable size of the parchment before cutting varies from 650-700 mm in the length of animal and 600-650 mm in the width of the animal. The length of the whole animal from neck along the spine to the tail could be up to 700-750 mm, representing rather young animals only a few weeks old or stillborn, in some cases, the maximum age could be a couple of months.

Borthwick Institute for Archives documents

Three of the six samples come from the collection of Morrell deeds held at the Borthwick Institute for Archives (MOR 10, MOR 30A, MOR 34), these are a varied collection of legal land documents from various sources, including auctions. The other three documents belong to the Yarburgh Family Archive (YM/D/ASK 12, YM/D/HAT 12, YM/D/CAMP 1), started in the 14th century this collection of documents has been held in a similar environment for most of its history and these documents have seldom been handled.

2. Biomolecular analysis

Parchment sampling

All sampling of the York Gospels and archival documents was completed using the dry non-invasive eraser based sampling technique of Fiddyment et al. (2015). 86 bifolia were sampled for protein analysis, a further eight bifolia and six archival documents were subjected to DNA analysis. For the DNA analysis samples with the greatest amount of starting material were utilised *circa* 150-250ul of eraser crumbs.

DNA extraction, library preparation and sequencing

DNA was extracted from the York Gospels and archival document samples using the protocol of Fiddyment *et al.* (2015) with a single modification: the use of eraser crumbs (*circa* 150-250ul) in place of cut parchment samples. Illumina sequencing libraries were produced for each of the samples and appropriate controls following the protocol of Meyer and Kircher (Meyer & Kircher, 2010), with modifications by Gamba *et al* (Gamba et al., 2014). An initial shotgun screen of the York Gospel libraries and controls only was completed on a single 65bp single end (SE) lane of an Illumina Miseq. The York Gospel samples and controls were then subsequently re-amplified and re-sequenced alongside the six archival documents on a further 150bp SE lane of an Illumina MiSeq. All sequencing was completed at TrinSeq, St James's Hospital, Dublin.

Read processing and analysis

Raw sequencing reads were trimmed of adapter sequences using cutadapt v1.11, with a minimum read length of 30bp maintained (Martin, 2011). A FastQ Screen (http://www.bioinformatics.babraham.ac.uk/projects/fastq_screen/) analysis was completed as an initial species assignment for each sample. For both sets of reads (65 and 150bp) two further alignments were then undertaken using BWA v0.7.13 (Li & Durbin, 2009) firstly, to the species used for the parchments production as detected by DNA and or eZooMS analysis (sheep (OviAri3) or cow (bosTau6)) and secondly to the human genome (hg19). The raw alignments were filtered using SAMtools v1.3 'rmdup' (Li et al., 2009).

Read filtering

The BAM files generated by the above alignments were further filtered for co-aligning endogenous or exogenous reads. In the case of the production species alignments (cow/sheep) any reads which also aligned to the human genome were removed. Conversely for the human genome(hg19) alignments any

reads that also aligned to the production species of that document (cow/sheep) were removed. Finally, to produce a "host filtered" dataset for metagenomic analysis via metaBIT the Cutadapt trimmed FASTQ files were filtered for reads that aligned to either the production species (cow/sheep) or the human genome.

DNA authenticity and damage assessment

A DNA damage and a read length assessment of the DNA sequences recovered from the parchment of the York Gospels was completed using mapDamage2.0 with standard settings on both the endogenous (cow or sheep) and exogenous (human) BAM files generated via the 150bp sequencing (Jónsson, Ginolhac, Schubert, Johnson, & Orlando, 2013) with default settings.

Sex determination

The sex of the animals that compose the York Gospels was deduced following the methods of Skoglund *et al. (2015).*

Population genetic analysis

Prior to population genetic analysis, the two sets of York Gospel filtered BAM files (65 and 150bp runs) were merged using Picard Tools (http://broadinstitute.github.io/picard/).

To provide a comparative dataset for the York Gospels samples a Bovine HD SNP dataset (770K) was downloaded from the WIDDE database (Sempéré et al., 2015). The ped and map files provided by WIDDE were then converted to geno and site files for use in LASER2.0 (Wang, Zhan, Liang, Abecasis, & Lin, 2015). The LASER2.0 pipeline (pileup2seq.py) was then used to convert the BAM files from the three York Gospel samples (Fol. 13, Fol. 101 and Fo. 125) with sufficient genome coverage into the LASER2.0 SEQ format and a LASER2.0 PCoA undertaken using default settings. The analysis was then visualized using R (R Core Team, 2017).

Metagenomic analysis

Two complementary metagenomic analyses were undertaken for each of the York Gospel samples the first using One Codex the second using metaBIT (Louvel, Der Sarkissian, Hanghøj, & Orlando, 2016). The One Codex analysis was completed using the trimmed FASTQ files with default settings including removal of host reads. The metaBIT analysis was completed using the trimmed host filtered FASTQ files with default settings, a pre-formatted HMP dataset provided by metaBIT was also analysed to provide a comparative analysis. Resulting datasets generated by metaBIT where then visualised in R (Albert & Yoder, 2013; R Core Team, 2017) .

STAMP analysis

The run_metaphlan2.pl script of Microbiome Helper (Comeau, Douglas, & Langille, 2017) was used to drive a MetaPhLan2 analysis on all the 150bp host filtered FASTQ files (n=15). The merged output file generated by run_metaphlan2.pl was then converted to STAMP format again using Microbiome Helper (metaphlan_to_stamp.pl). STAMP was then used to complete both a PCoA analyses and an analysis of significantly differentiated taxa between the samples at the genus level, using default settings.

3. Supplementary Figures

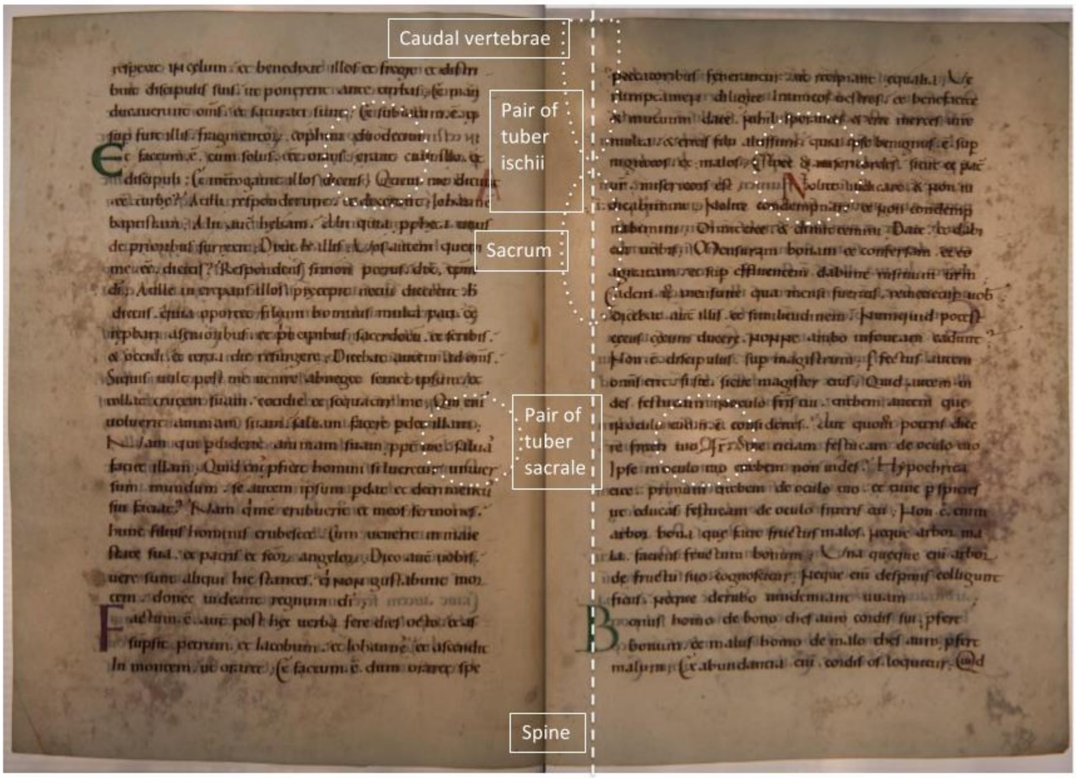

Supplementary figure 1: The York Gospels bifolium 101v / 96r in transmitting light (virtual reconstruction of conjoined folia) shows clearly the pelvis, sacrum and first vertebrae of the tail of the calf. The spine of the animal is in the vertical position (parallel with the spine of the book) indicating that only two bifolia could be made from one skin. This is a typical layout for 'luxury manuscripts' when only the best parts of the parchment were selected for production of manuscripts. Image reproduced by kind permission of the Chapter of York.

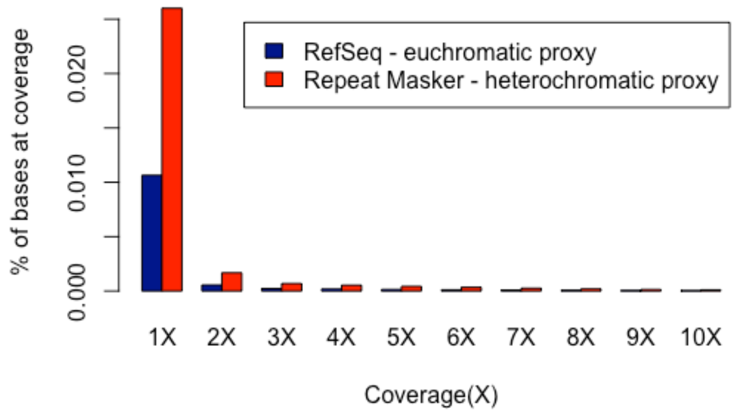

Supplementary figure 2: Comparison of percentage genome coverage for Folio 125 in proxies for euchromatin (UCSC RefSeq track) and heterochromatic regions (UCSC RepeatMasker track). The greater relative coverage of the RepeatMasker track may reflect preferential conservation of heterochromatin in parchment samples due to the harsh alkaline treatment inherent in parchments production.

a)

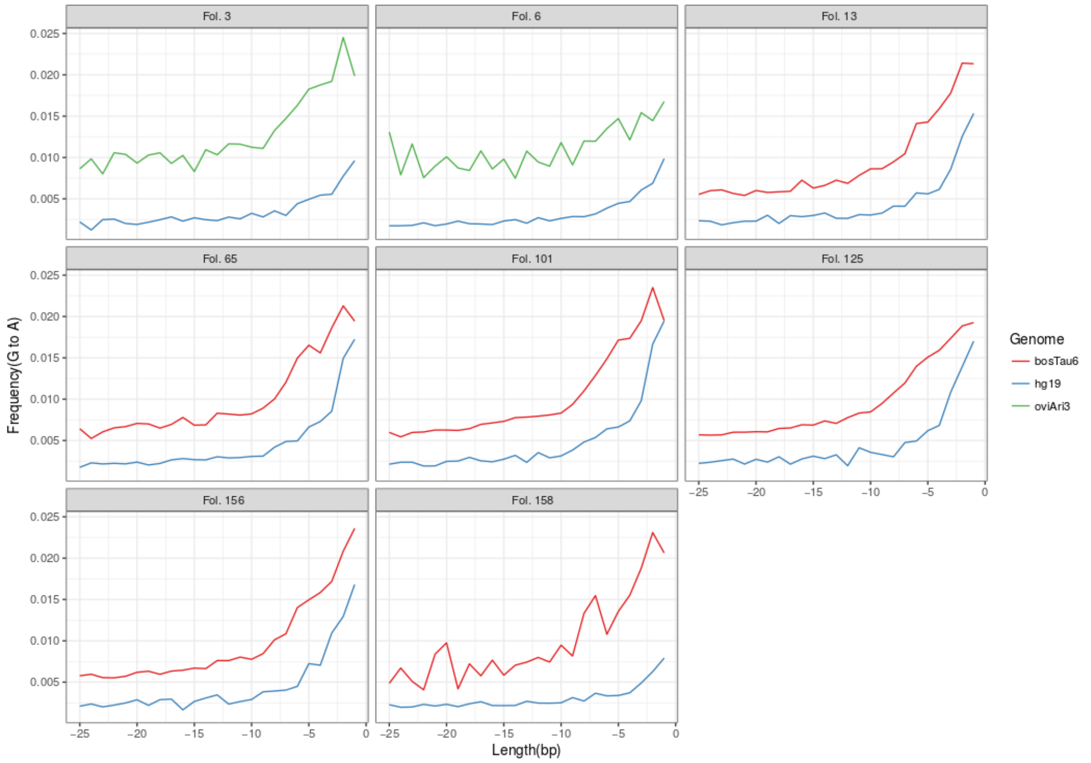

b)

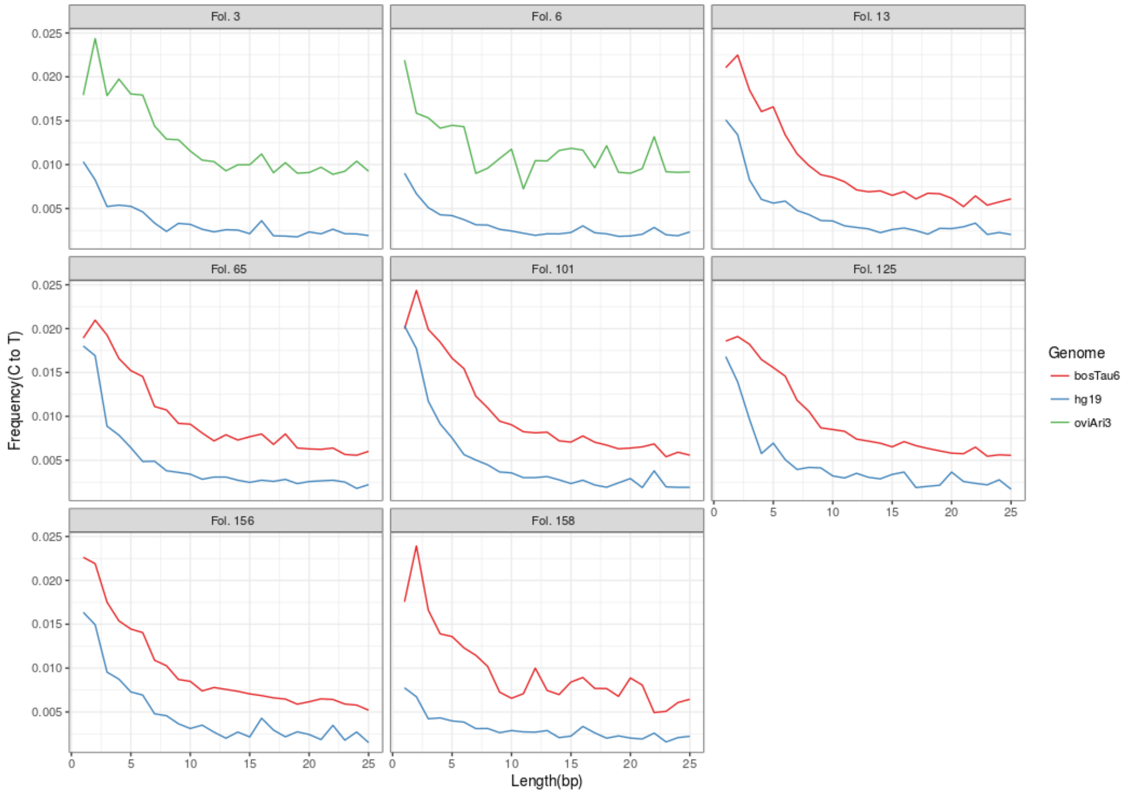

Supplementary figure 3: Comparison of DNA damage patterns at the a = 5' and b =3' ends of York Gospel parchment sequences. Limited DNA damage is seen for each sample, with the endogenous DNA sequences showing a relatively greater level of deamination.

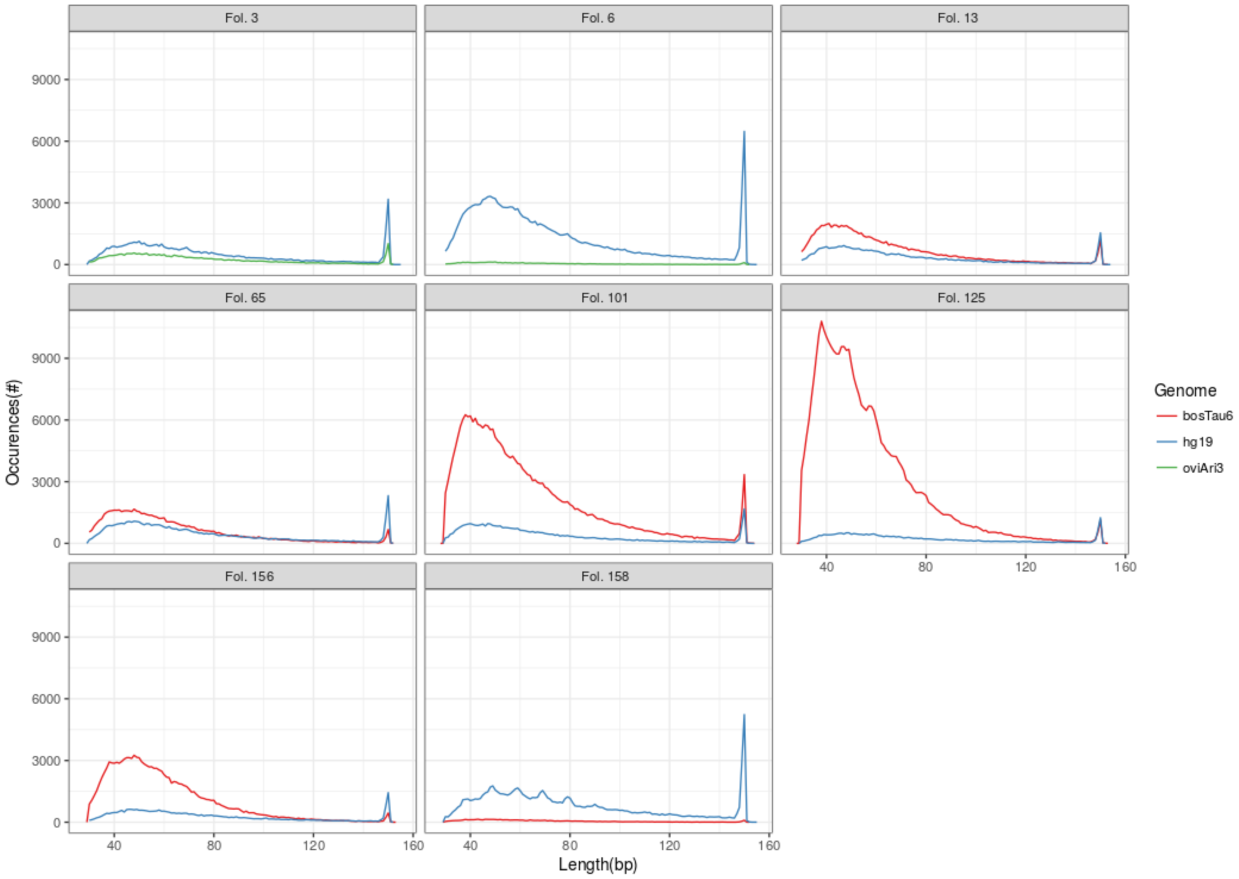

Supplementary figure 4: Read lengths of exogenous (human = hg19) and endogenous DNA (sheep = oviAri3 or cow = bosTau6) sequences recovered from the York Gospels (150bp SE reads). The read length distribution of the exogenous DNA fragments in YG28 suggests consistency with histone wrapping.

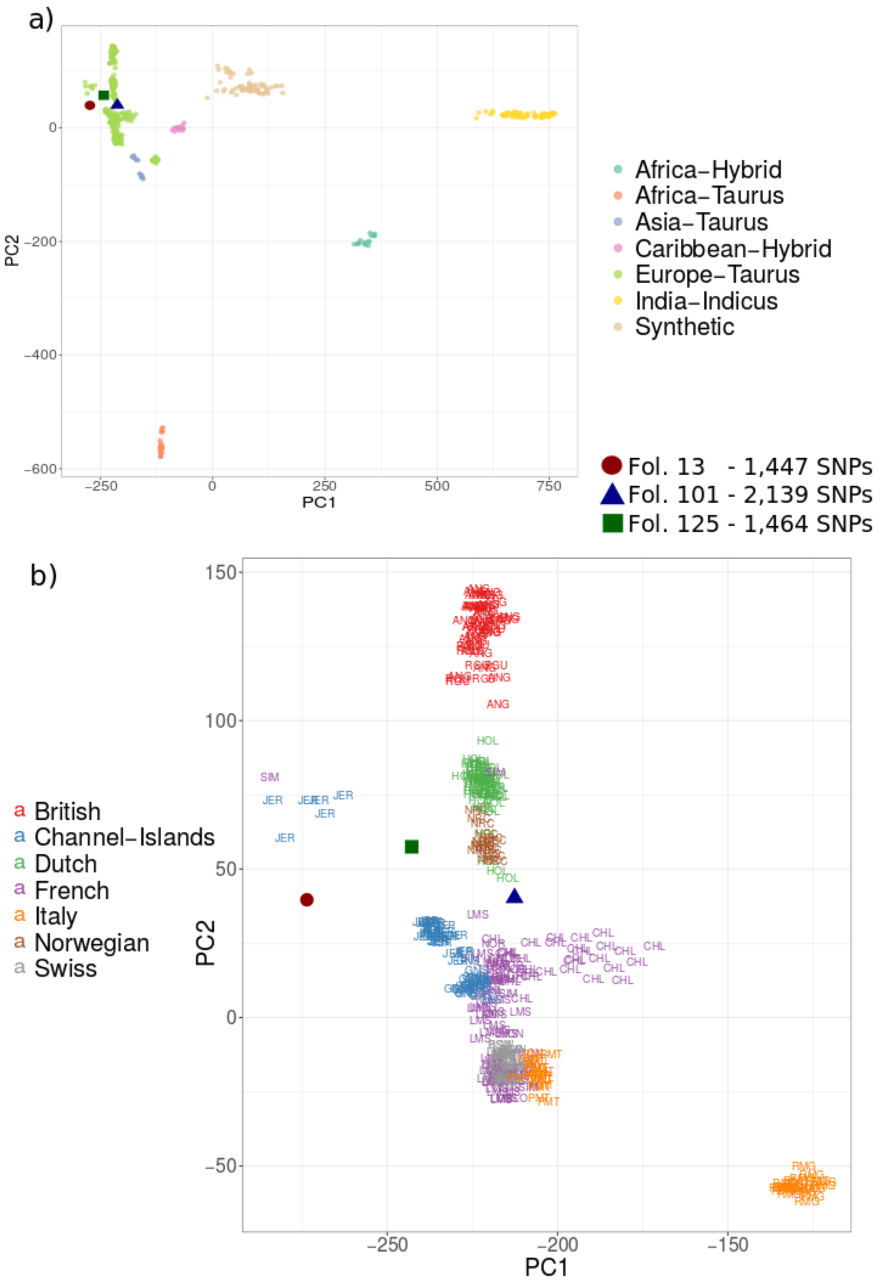

Supplementary figure 5: LASER2.0 PCoA of DNA sequences recovered from three folio of the York Gospels, all Gospel samples are seen to show a genetic affinity to bovine reference populations (WIDDE) sampled in Europe. **a)** Global dataset. **b)** European breeds only, colours represent the breeds proposed country of origin, 3 letter breed codes are used for plotting (ANG=Angus, BLO=Blonde d'Aquitaine, BSW=Brown Swiss, CHL=Charolais, GNS=Guernsey, HOL=Holstein, JER=Jersey, LMS=Limousin, MON=Montbeliarde, NOR=Normande, NRC=Norwegian Red Cattle, PMT=Piedmontese, RGU=Red Angus, RMG=Romagnola, SIM=Simmental).

a)

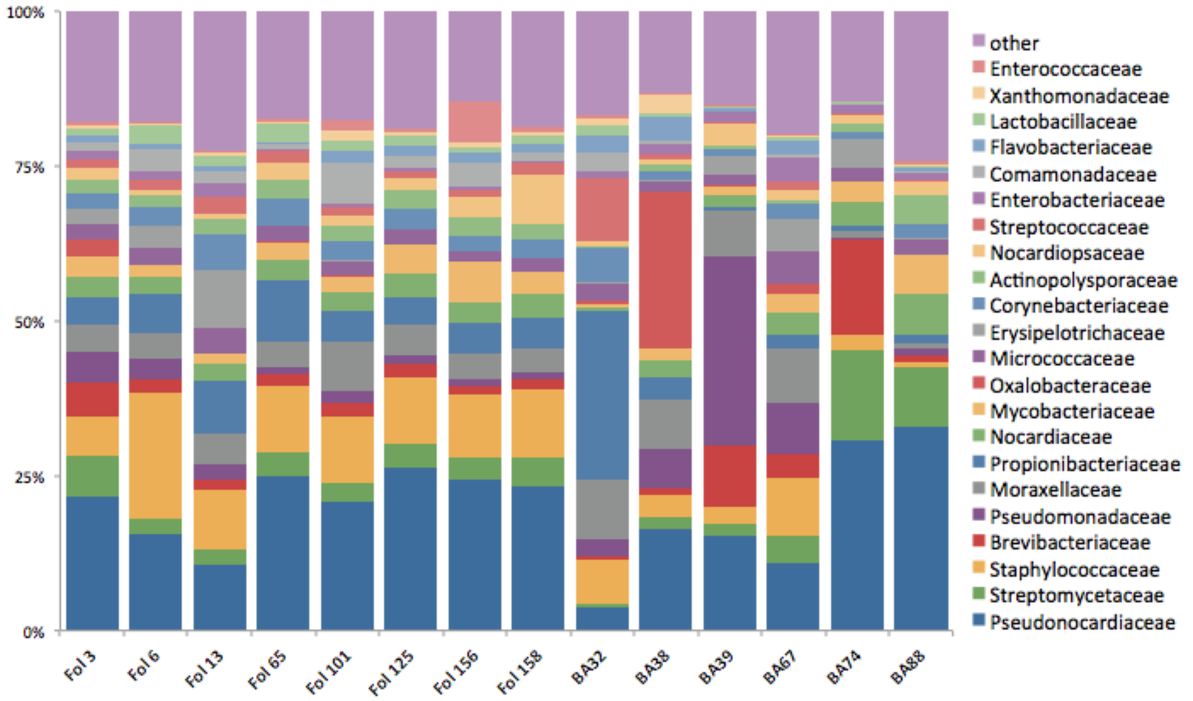

b)

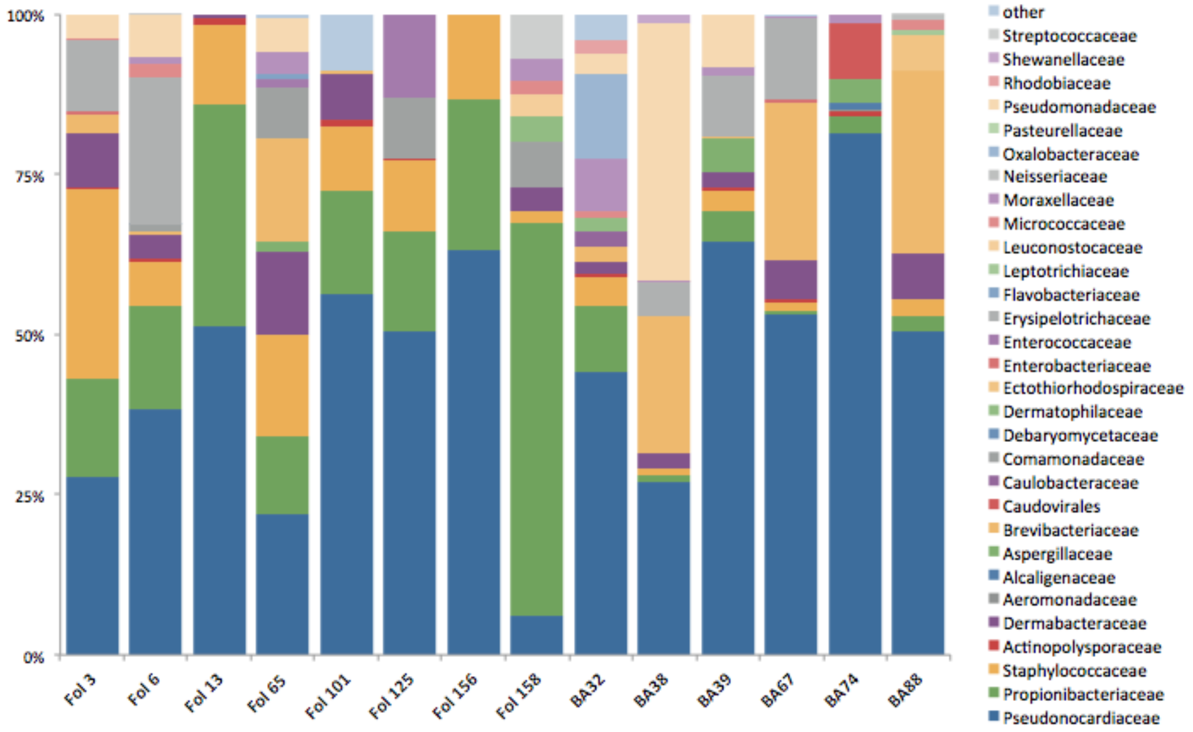

Supplementary figure 6: a) Taxonomic profiles from One Codex with identification set to family level based on at least 3% reads being identified to a particular family (human reads removed). **b)** Taxonomic data extracted from metaBIT pipeline (150bp, human reads removed) identified to Family level.

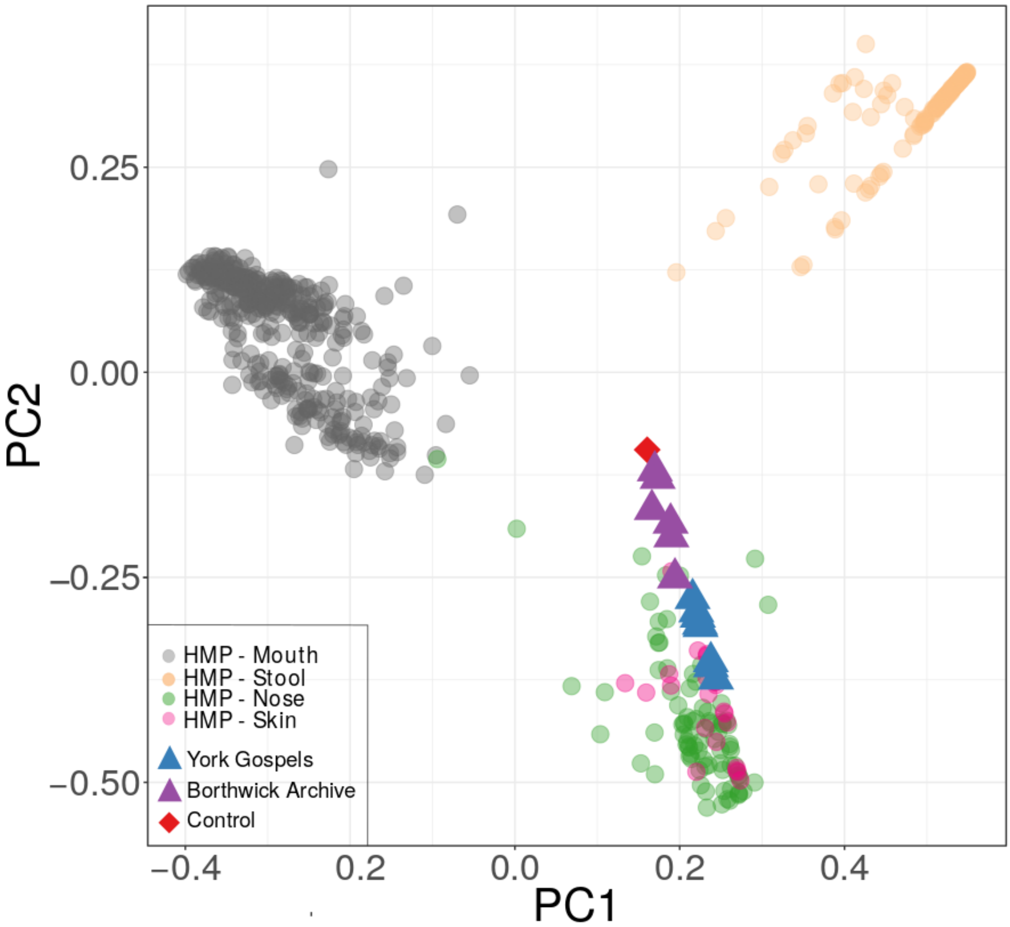

Supplementary figure 7: metaBIT PCoA analysis of York Gospel and Borthwick Archive samples (Genera) 150bp reads with extraction control, host reads removed. HMP dataset provided by metaBIT.

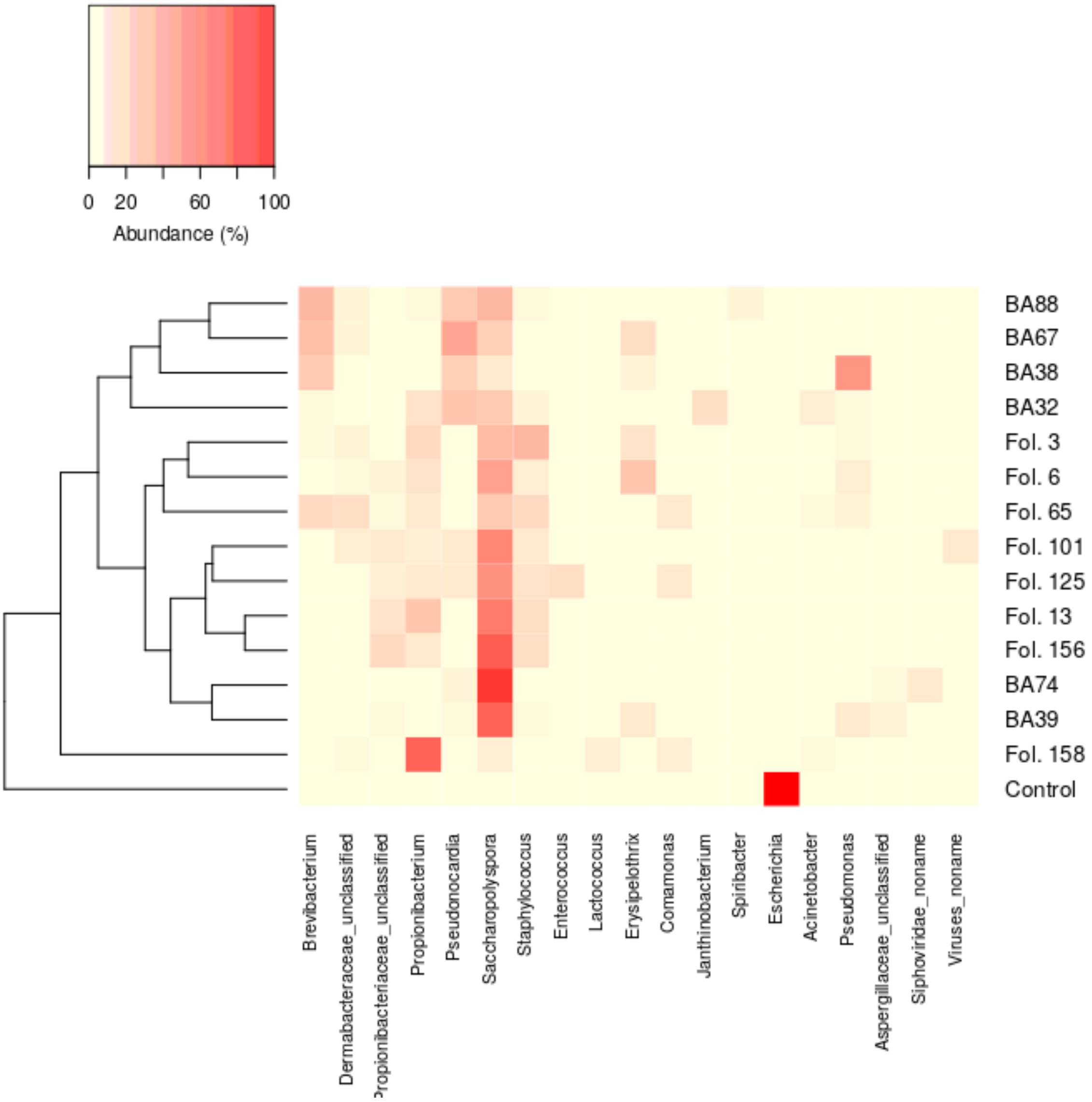

Supplementary figure 8: Heatmap of identified genera recovered from the York Gospel samples via metaBIT with extraction control, utilising the 150bp dataset. Genera shown represent >5% abundance in at least one sample. Clustering of samples (dendrogram) was completed using the complete metaBIT filtered genus output (n=29)

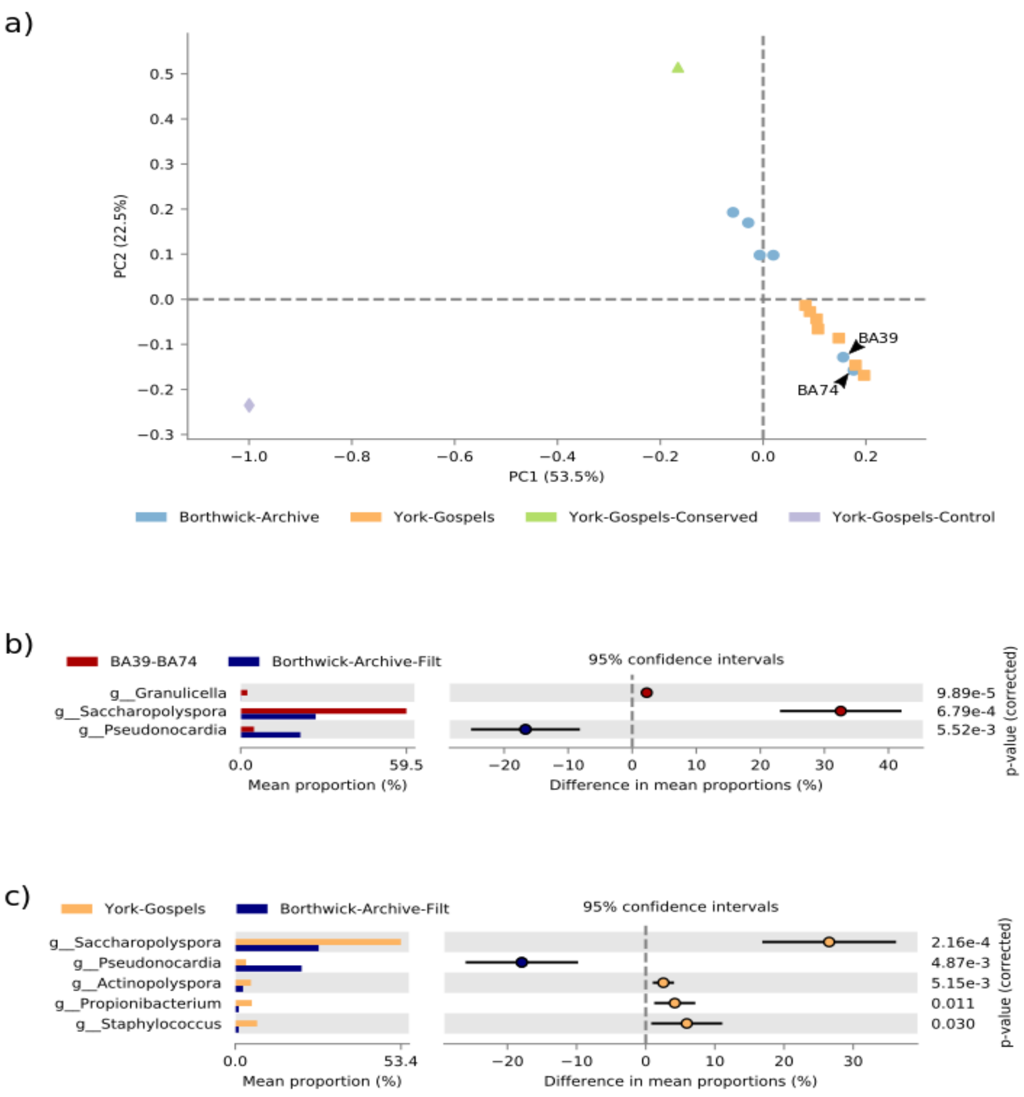

Supplementary figure 9: STAMP analysis, **a)** PCA of taxonomic profiles generated by MetaPhlAn2 from the 150bp SE read parchment data at genus level. **b)** Analysis of significantly differentiated species between BA39 and BA74 and the remainder of Borthwick Archive samples (Welch's t-test; p-value > 0.05), at genus level. **c)** Analysis of significantly differentiated species between the filtered Borthwick Archive samples (no BA39 or BA74) vs. unconserved York Gospels (Welch's t-test; p-value > 0.05), at genus level.

4. Supplementary Tables

Supplementary table 1. Table of all samples analysed using eZooMS

Sample Number	Start Date of Document	End Date of Document	Provenance (Town)	Type of Document	Area sampled	Species ID
YG 29	1300	1600	Yorkshire		flyleaf fol 2 recto	Sheep
YG 30	1300	1600	Yorkshire	Oath of a proxy for an archdeacon	3 r	Sheep
YG 31	1300	1600	Yorkshire	Oaths of a subdean in person, a canon and prebendary in person, and an archdeacon	4 r	Sheep
YG 32	1300	1600	Yorkshire	Oaths of a subdean in person, a canon and prebendary in person, and an archdeacon	8 r	Sheep
YG 33	1300	1600	Yorkshire	Oaths of a subdean in person, a canon and prebendary in person, and an archdeacon	9r single	Sheep
YG 34	990	1020	Canterbury	Gospels	14 v	Calf
YG 35	990	1020	Canterbury	Gospels	13 v	Calf
YG 36	990	1020	Canterbury	Gospels	12 r	Calf
YG 37	990	1020	Canterbury	Gospels	15 r	Calf
YG 38	990	1020	Canterbury	Gospels	16 r	Calf
YG 39	990	1020	Canterbury	Gospels	17 r	Calf
YG 40	990	1020	Canterbury	Gospels	18 r	Calf
YG 41	990	1020	Canterbury	Gospels	27r	Calf
YG 42	990	1020	Canterbury	Gospels	28 r	Calf
YG 43	990	1020	Canterbury	Gospels	29 r	Calf
YG 44	990	1020	Canterbury	Gospels	30 r	Calf
YG 45	990	1020	Canterbury	Gospels	35 r	Calf
YG 46	990	1020	Canterbury	Gospels	36 r	Calf
YG 47	990	1020	Canterbury	Gospels	37 r	Calf
YG 48	990	1020	Canterbury	Gospels	38 r	Calf
YG 49	990	1020	Canterbury	Gospels	42 r	Calf
YG 50	990	1020	Canterbury	Gospels	41 r	Calf
YG 51	990	1020	Canterbury	Gospels	40 r	Calf
YG 52	990	1020	Canterbury	Gospels	39 v	Calf
YG 53	990	1020	Canterbury	Gospels	54 r	Calf
YG 54	990	1020	Canterbury	Gospels	53 r	Calf
YG 55	990	1020	Canterbury	Gospels	52 r	Calf
YG 56	990	1020	Canterbury	Gospels	51 r	Calf
YG 57	990	1020	Canterbury	Gospels	58 v	Calf
YG 58	990	1020	Canterbury	Gospels	57 v	Calf
YG 59	990	1020	Canterbury	Gospels	56 v	Calf
YG 60	990	1020	Canterbury	Gospels	55 v	Calf
YG 61	990	1020	Canterbury	Gospels	70 v	Calf
YG 62	990	1020	Canterbury	Gospels	69 v	Calf
YG 63	990	1020	Canterbury	Gospels	68 v	Calf

YG 64	990	1020	Canterbury	Gospels	67 v	Calf
YG 65	990	1020	Canterbury	Gospels	75 r	Calf
YG 66	990	1020	Canterbury	Gospels	76 r	Calf
YG 67	990	1020	Canterbury	Gospels	77 r	Calf
YG 68	990	1020	Canterbury	Gospels	78 r	Calf
YG 69	990	1020	Canterbury	Gospels	82 r	Calf?
YG 70	990	1020	Canterbury	Gospels	81 r	Calf?
YG 71	990	1020	Canterbury	Gospels	80 r	Calf
YG 72	990	1020	Canterbury	Gospels	79 r	Calf
YG 73	990	1020	Canterbury	Gospels	84 r	Sheep
YG 74	990	1020	Canterbury	Gospels	92 r	Calf
YG 75	990	1020	Canterbury	Gospels	93 v	Calf
YG 76	990	1020	Canterbury	Gospels	94 v	Calf
YG 77	990	1020	Canterbury	Gospels	91 v	Calf
YG 78	990	1020	Canterbury	Gospels	99 v	Calf
YG 79	990	1020	Canterbury	Gospels	100 v	Calf
YG 80	990	1020	Canterbury	Gospels	101 v	Calf
YG 81	990	1020	Canterbury	Gospels	102 r	Calf
YG 82	990	1020	Canterbury	Gospels	107 r	Calf
YG 83	990	1020	Canterbury	Gospels	108 r	Calf
YG 84	990	1020	Canterbury	Gospels	109 r	Calf
YG 85	990	1020	Canterbury	Gospels	110 r	Calf
YG 86	990	1020	Canterbury	Gospels	115 r	Calf
YG 87	990	1020	Canterbury	Gospels	116 r	Calf
YG 88	990	1020	Canterbury	Gospels	117 r	Calf
YG 89	990	1020	Canterbury	Gospels	118 r	Calf
YG 90	990	1020	Canterbury	Gospels	119 r	Calf
YG 91	990	1020	Canterbury	Gospels	120 r	Calf
YG 92	990	1020	Canterbury	Gospels	121 r	Calf
YG 93	990	1020	Canterbury	Gospels	122 r	Calf
YG 94	990	1020	Canterbury	Gospels	129 r	Calf
YG 95	990	1020	Canterbury	Gospels	130 r	Calf
YG 96	990	1020	Canterbury	Gospels	131 r	Calf
YG 97	990	1020	Canterbury	Gospels	132 v	Calf
YG 98	990	1020	Canterbury	Gospels	133 v	Calf
YG 99	990	1020	Canterbury	Gospels	134 v	Calf
YG 100	990	1020	Canterbury	Gospels	135 r	Calf
YG 101	990	1020	Canterbury	Gospels	140 r	Calf
YG 102	990	1020	Canterbury	Gospels	141 v	Calf
YG 103	990	1020	Canterbury	Gospels	142 v	Calf
YG 104	990	1020	Canterbury	Gospels	143 v	Calf
YG 105	990	1020	Canterbury	Gospels	152 r	Calf
YG 106	990	1020	Canterbury	Gospels	153 v	Calf
YG 107	990	1020	Canterbury	Gospels	154 r	Calf
YG 108	990	1020	Canterbury	Gospels	155 r	Calf
YG 109	990	1020	Canterbury	Main text of the Gospels (Canterbury?) and survey of archiepiscopal property at Sherburn-in-Elemet (Yorkshire)	156 r	Calf

YG 110	1020	1020	Canterbury	Surveys of archiepiscopal property at Otley and Ripon	157 r	Calf
YG 111	1020	1023	Canterbury	Homily by Wulfstan	158 r	Calf?
YG 112	990	1020	Canterbury	Gospels	159 r	Calf
YG 113	1025	1100	Yorkshire	Inventory of church goods at Sherburn-in-Elmet, 'Bidding of Prayer', and List of sureties of AElfric	162 v	No ID
YG 114	1500	1600	Yorkshire	Inventory of church goods at Sherburn-in-Elmet, 'Bidding of Prayer', and List of sureties of AElfric	163 v	No ID
YG 115	1500	1600	Yorkshire	Blank except for signature, 'Joh(ann)es Caerlyle'	166 r	No ID
BA32	1455	1455	Yorkshire	Legal Document (MOR10)	N/A	Sheep
BA38	1723	1723	Yorkshire	Legal Document (MOR30A)	N/A	Sheep
BA39	1648	1648	Yorkshire	Legal Document (MOR34)	N/A	Sheep
BA67	1556	1556	Yorkshire	Legal Document (YM.D/ASK12)	N/A	Sheep
BA74	1812	1812	Yorkshire	Legal Document (YM/D/H AT 12)	N/A	Sheep
BA88	1322	1322	Yorkshire	Legal Document (YM/D/CAMP 1)	N/A	Sheep

Folio number	Lab Code	DNA concentration [ng/ul]	Total Raw Reads	Final Aligned Reads+	Final Aligned Reads+ (%)	Species ID (proteomics)	Species ID (DNA)	Sex	Diversity index
Fol. 3	YG01	6.5	2,586,155	48,232	1.9	Sheep	?	Inconclusive (0.757)	1.7459
Fol. 6	YG13	1.4	3,209,492	7,995	0.2	Sheep	?	Inconclusive (0.72)	1.7583
Fol. 13	YG14	2.6	2,675,173	110,317	4.1	Cow	Cow	Male (0.562)	1.0487
Fol. 65	YG15	2.4	2,366,689	50,525	2.1	Cow	Cow	Female (0.903)	2.1077
Fol. 101	YG16	2.0	3,577,046	203,226	5.7	Cow	Cow	Female (1.019)	1.2116
Fol. 125	YG17	1.7	2,719,891	152,910	5.6	Cow	Cow	Female (0.96)	1.5855
Fol. 156	YG18	7.7	2,082,259	59,763	2.9	Cow	Cow	Female (1.009)	0.8986
Fol. 158	YG28	0.0	2,117,610	6,921	0.3	Cow?	Cow?	Inconclusive (0.675)	1.4855

Supplementary table 2: Sample information and alignment statistics for the samples of the York Gospels, +Duplicate reads removed (SAMtools rmdup) and human reads removed; mapping quality >= 30. Sex indetification following Skoglund *et al* 2015, Diversity index from metaBIT output.

	65bp					150bp					Merged				
Sample	Raw Reads	Raw Alignment	hg19 Reads Removed	After rmdup	Final Aligned Reads^{+} (%)	Raw Reads	Raw Alignment	hg19 Reads Removed	After rmdup	Final Aligned Reads^{+} (%)	Total Reads	Final Aligned Reads^{+}	Final Aligned Reads^{+} (%)	Mapping Quality 30	Mapping Quality 30 (%)
Fol. 3	1,411,830	89,707	85,521	78,176	5.5	1,174,325	67,405	64,615	59,649	5.1	2,586,155	137,825	5.3	48,232	1.9
Fol. 6	1,603,904	17,767	11,511	11,352	0.7	1,605,588	15,651	10,346	10,219	0.6	3,209,492	21,571	0.7	7,995	0.2
Fol. 13	1,779,563	347,388	339,177	337,069	18.9	895,610	168,562	165,030	164,506	18.4	2,675,173	501,575	18.7	110,317	4.1
Fol. 65	1,372,548	212,001	206,392	205,382	15.0	994,141	147,124	143,706	143,170	14.4	2,366,689	348,552	14.7	50,525	2.1
Fol. 101	2,119,311	806,007	796,196	783,629	37.0	1,457,735	528,594	522,702	516,755	35.4	3,577,046	1,300,384	36.4	203,226	5.7
Fol. 125	1,308,579	699,177	692,822	678,670	51.9	1,411,312	740,908	734,649	719,143	51.0	2,719,891	1,397,813	51.4	152,910	5.6
Fol. 156	1,069,640	283,815	280,011	277,925	26.0	1,012,619	264,487	261,244	259,297	25.6	2,082,259	537,222	25.8	59,763	2.9
Fol. 158	1,004,636	22,541	11,828	11,793	1.2	1,112,974	21,926	12,679	12,647	1.1	2,117,610	24,440	1.2	6,921	0.3

Supplementary table3: Raw alignment data from the York Gospels, $^{+}$SAMtools 'rmdup', hg19 reads removed.

Sample	Raw Reads	Aligned reads raw	Aligned reads raw (%)
BA32R	1,593,039	204,035	12.8
BA38	1,064,851	32,845	3.1
BA39	1,327,754	88,301	6.7
BA67	2,047,706	78,405	3.8
BA74	2,626,299	49,725	1.9
BA88	837,889	46,009	5.5
Fol. 3	1,174,325	123,529	10.5
Fol. 6	1,605,588	333,479	20.8
Fol. 13	895,610	88,752	9.9
Fol. 65	994,141	112,459	11.3
Fol. 101	1,457,735	98,475	6.8
Fol. 125	1,411,312	59,815	4.2
Fol. 156	1,012,619	70,968	7.0
Fol. 158	1,112,974	207,028	18.6

Supplementary table 4: Human genome alignment statistics for the York Gospels and Borthwick Archive samples, 150bp reads, PCR duplicates removed with SAMtools 'rmdup'.

Sample	Total Reads	Final Aligned Reads+	Final Aligned Reads+ (%)	Mapping quality 30	Mapping quality 30 (%)
Fol. 3	2,586,155	268,527	10.4	227,953	8.8
Fol. 6	3,209,492	662,114	20.6	561,539	17.5
Fol. 13	2,675,173	257,290	9.6	216,517	8.1
Fol. 65	2,366,689	261,246	11.0	221,496	9.4
Fol. 101	3,577,046	230,713	6.4	193,941	5.4
Fol. 125	2,719,891	104,760	3.9	89,231	3.3
Fol. 156	2,082,259	141,437	6.8	121,183	5.8
Fol. 158	2,117,610	378,222	17.9	323,255	15.3

Supplementary table 5: Filtered Human genome alignment statistics for the York Gospels all reads. +Duplicate reads removed (SAMtools rmdup) and endogenous (cow/sheep) reads removed.

5. References

Albert, A., & Yoder, J. (2013, August 20). Making heatmaps with R for microbiome analysis. Retrieved June 7, 2017, from http://www.molecularecologist.com/2013/08/making-heatmaps-with-r-for-microbiome-analysis/

Comeau, A. M., Douglas, G. M., & Langille, M. G. I. (2017). Microbiome Helper: a Custom and Streamlined Workflow for Microbiome Research. *mSystems*, *2*(1). https://doi.org/10.1128/mSystems.00127-16

Fiddyment, S., Holsinger, B., Ruzzier, C., Devine, A., Binois, A., Albarella, U., … Collins, M. J. (2015). Animal origin of 13th-century uterine vellum revealed using noninvasive peptide fingerprinting. *Proceedings of the National Academy of Sciences of the United States of America*, *112*(49), 15066–15071.

Gamba, C., Jones, E. R., Teasdale, M. D., McLaughlin, R. L., Gonzalez-Fortes, G., Mattiangeli, V., … Pinhasi, R. (2014). Genome flux and stasis in a five millennium transect of European prehistory. *Nature Communications*, *5*. https://doi.org/10.1038/ncomms6257

Jónsson, H., Ginolhac, A., Schubert, M., Johnson, P. L. F., & Orlando, L. (2013). mapDamage2.0: fast approximate Bayesian estimates of ancient DNA damage parameters. *Bioinformatics* , *29*(13), 1682–1684.

Li, H., & Durbin, R. (2009). Fast and accurate short read alignment with Burrows-Wheeler transform. *Bioinformatics* , *25*(14), 1754–1760.

Li, H., Handsaker, B., Wysoker, A., Fennell, T., Ruan, J., Homer, N., … 1000 Genome Project Data Processing Subgroup. (2009). The Sequence Alignment/Map format and SAMtools. *Bioinformatics* , *25*(16), 2078–2079.

Louvel, G., Der Sarkissian, C., Hanghøj, K., & Orlando, L. (2016). metaBIT, an integrative and automated metagenomic pipeline for analyzing microbial profiles from high-throughput sequencing shotgun data. *Molecular Ecology Resources*. https://doi.org/10.1111/1755-0998.12546

Martin, M. (2011). Cutadapt removes adapter sequences from high-throughput sequencing reads. *EMBnet.journal*, *17*(1), 10–12.

Meyer, M., & Kircher, M. (2010). Illumina Sequencing Library Preparation for Highly Multiplexed Target Capture and Sequencing. *Cold Spring Harbor Protocols*, *2010*(6), db.prot5448–pdb.prot5448.

R Core Team. (2017). R: A Language and Environment for Statistical Computing. Vienna, Austria: R Foundation for Statistical Computing. Retrieved from https://www.R-project.org/

Sempéré, G., Moazami-Goudarzi, K., Eggen, A., Laloë, D., Gautier, M., & Flori, L. (2015). WIDDE: a Web-Interfaced next generation database for genetic diversity exploration, with a first application in cattle. *BMC Genomics*, *16*(1), 940.

Skoglund, P., Ersmark, E., Palkopoulou, E., & Dalén, L. (2015). Ancient Wolf Genome Reveals an Early Divergence of Domestic Dog Ancestors and Admixture into High-Latitude Breeds. *Current Biology: CB*. https://doi.org/10.1016/j.cub.2015.04.019

Wang, C., Zhan, X., Liang, L., Abecasis, G. R., & Lin, X. (2015). Improved Ancestry Estimation for both Genotyping and Sequencing Data using Projection Procrustes Analysis and Genotype Imputation. *American Journal of Human Genetics*. https://doi.org/10.1016/j.ajhg.2015.04.018
